# Comparison of preoperative and intraoperative cultures for predicting postoperative urinary tract infections following supine PCNL

**DOI:** 10.1007/s00345-026-06320-5

**Published:** 2026-03-02

**Authors:** Gunal Ozgur, Dogancan Dorucu, Orhan Bugra Duran, Ersin Gokmen, Yusuf Senoglu, Haydar Kamil Cam, Tarik Emre Sener

**Affiliations:** 1 Department of Urology, Marmara University Pendik Training and Research Hospital, 34890 Istanbul, Turkey; 2 Department of Urology, Siverek State Hospital, Sanliurfa, Turkey; 3 Department of Urology, Marmara University School of Medicine, Istanbul, Turkey

**Keywords:** Complications, Kidney calculi, Percutaneous nephrolithotomy, Urinary tract infections, Urine culture

## Abstract

**Introduction:**

This study aimed to compare the predictive value of preoperative midstream urine culture (PMUC), intraoperative renal pelvic urine culture (RPUC), and stone culture (SC) for postoperative urinary tract infections (UTIs) following percutaneous nephrolithotomy (PCNL).

**Methods:**

We retrospectively analyzed 234 patients who underwent supine-PCNL between January 2020 and April 2025. UTI was diagnosed based on systemic inflammatory response syndrome criteria and elevated inflammatory markers. Demographic, peri-, intra- and post-operative data were compared between patients with and without UTI. Multivariate logistic regression identified independent predictors.

**Results:**

UTI occurred in 14.1%(*n* = 33) of patients postoperatively, with 72.7% presenting with fever. Culture positivity rates were significantly higher in postoperative UTI-patients (PMUC = 27.3% vs. 7.5%, SC: 39.4% vs. 8.0% and RPUC: 30.3% vs. 6.0%; *p* < 0.001). In UTI-patients, only 15.2% of postoperative urine cultures obtained before antibiotic treatment showed bacterial growth, which was lower than intraoperative cultures. UTI was higher in female patients (60.6% vs. 39.4%) and in those with an ASA score of 3 (*p* = 0.001 and *p* = 0.020). Female gender (OR = 3.71, *p* = 0.004), ASA-3 score (OR = 5.13, *p* = 0.029), positive SC (OR = 5.83, *p* = 0.001), and RPUC (OR = 3.67, *p* = 0.023) were independent predictors of postoperative UTI. PMUC was not associated (*p* = 0.65) with postoperative UTI in the multivariate analysis.

**Conclusions:**

Intraoperative SC and RPUC showed a stronger association with postoperative UTI compared with PMUC and may be considered for routine use. Female gender and ASA-3 score were identified as independent risk factors. In patients who develop UTI, prior empirical or prophylactic antibiotic use may limit pathogen detection in postoperative urine cultures; therefore, intraoperative cultures play a critical role in early and targeted treatment.

## Introduction

Percutaneous nephrolithotomy (PCNL) is the first-line option for treatment of large renal stones (> 2 cm) [1]. Although PCNL has a very low complication rate, postoperative infections represent a significant concern. Postoperative fever occurs in approximately 10.8% of patients, while sepsis is reported in about 0.5% [2]. Antibiotic prophylaxis combined with a sterile preoperative midstream urine culture (PMUC) has been shown to reduce the risk of postoperative fever and related complications [3]. Nevertheless, infectious complications may still occur in the postoperative period despite these precautions, and devastating outcomes due to urosepsis may be experienced [4, 5].

Bacterial colonization within kidney stones and increased intrapelvic pressure during surgery are thought to contribute to the development of postoperative infectious complications [1, 6]. Given that kidney stones may serve as a source of infection, intraoperative renal pelvic urine culture (RPUC) and stone culture (SC) have been reported to be more reliable than PMUC in identifying the causative microorganisms [7]. These intraoperative cultures are considered particularly important for the early and effective management of post-operative infectious events. The European Association of Urology (EAU) guidelines also emphasize that SC or urine culture obtained directly from the renal pelvis provide greater predictive value for postoperative sepsis compared to PMUC [1].

Several studies have examined the associations between PMUC, intraoperative RPUC and SC with infectious outcomes following PCNL [8]. However, studies comparing all three culture types within the same patient cohort are limited. Moreover, some discrepancies exist among the results of these studies [9].

The primary aim of this study was to compare the predictive value of preoperative PMUC, intraoperative RPUC, and SC for postoperative UTIs following supine-PCNL. The secondary aim was to identify patient-, stone-, and procedure-related factors associated with postoperative UTIs, including independent predictors determined through multivariate analysis.

## Materials and methods

This study was approved by the Institutional Review Board (Approval No: 09.2025.25–0192) and conducted in accordance with the principles of the Declaration of Helsinki. A retrospective analysis was performed on patients who underwent supine PCNL for kidney stones between January 2020 and April 2025. Patients were included if they had available PMUC and intraoperative RPUC and SC. Exclusion criteria included simultaneous bilateral endoscopic surgery (SBES), anatomical or functional urinary tract anomalies (e.g., ureteropelvic junction obstruction, horseshoe kidney, vesicoureteral reflux), diagnosis of immunodeficiency, or age under 18 years.

In accordance with the recommendations of the EAU guidelines, a PMUC was obtained from all patients included in the study [1]. For those with sterile cultures, antibiotic prophylaxis was determined in collaboration with the institution’s infectious diseases committee, taking into account local antimicrobial susceptibility patterns [10]. Patients with sterile urine cultures received a single dose of 2 g ceftriaxone administered within 30 min prior to the surgical incision. In patients with positive urine cultures, antibiotic treatment was initiated based on antimicrobial susceptibility testing and continued for a minimum of seven days, as recommended by the infectious diseases specialists. Once sterile cultures were achieved, these patients underwent surgery with the appropriate prophylactic antibiotic regimen as guided by infectious diseases consultation. In statistical analysis, these patients were classified as PMUC positive.

All PCNL procedures were performed in the supine Galdakao-Modified Valdivia position by experienced endourologists. During the operation, urine sample was obtained directly from the renal pelvis for RPUC. Following stone fragmentation, stone fragments were collected for SC. In the postoperative period, patients who developed systemic inflammatory response syndrome (SIRS; defined as having two or more of the following: temperature < 36 °C or > 38 °C, heart rate > 90 bpm, respiratory rate > 20 breaths per minute, or white blood cell count < 4000/mm³ or > 12000/mm³) and showed elevations in acute phase reactants (C-reactive protein, procalcitonin), were evaluated by the Infectious Diseases Department. Patients diagnosed with infection received appropriate antibiotic regimen and these patients were classified as positive for UTI. Patients were divided into two groups based on the presence or absence of post-operative UTI.

Within the scope of the study, demographic data, stone characteristics, pre- and peri-operative surgical parameters, and postoperative clinical outcomes were evaluated. In addition, the results of PMUC, as well as intraoperative SC and RPUC, were analyzed. All parameters were compared between patients with and without post-operative UTI to determine potential associations.

## Statistical analysis

All analyses were performed using IBM SPSS Statistics version 25. Normality of variables was assessed via visual (histograms, probability plots) and analytical methods (Kolmogorov–Smirnov/Shapiro–Wilk tests). Descriptive statistics were expressed as mean (± standard deviation: SD) for normally distributed variables and median (interquartile range: IQR) for non-normally distributed ones. Group comparisons were made using the independent t-test or Mann–Whitney U test, depending on distribution. Categorical variables were compared using Chi-square or Fisher’s exact test where appropriate. Multivariate logistic regression was performed to identify independent predictors of postoperative UTI, including factors with *p* < 0.25 in univariate analysis. A p-value < 0.05 was considered statistically significant.

## Results

A total of 234 patients were included in the study. The incidence of postoperative UTI was 14.1% (*n* = 33). Postoperative early fever was detected in 29 patients (12.4%). While only 3% of patients without postoperative UTI had fever, the incidence was markedly higher at 72.7% in the UTI-positive group. Five patients (2.14%) required intensive care unit monitoring due to urosepsis. One elderly patient (0.4%) with cardiac and neurological comorbidities developed a renal hematoma and sepsis after surgery and died while being monitored in the intensive care unit.

There were no significant differences between UTI-positive and UTI-negative patients in terms of age, BMI, presence of diabetes, stone size, or laterality of surgery (*p* > 0.05). However, the proportion of female patients was significantly higher in the UTI-positive group (60.6% vs. 39.4%; *p* = 0.001). In addition, the occurrence of UTI was more common among patients with an ASA score of 3 (*p* = 0.020) (Table 1).

**Table 1:** Baseline Demographics and Stone Characteristics by UTI Status

Variables	UTI (-) *N* = 201 (85.9%)	UTI (+) *N* = 33 (14.1%)	*P* value
Age, years	46.8 (± 16.3)	48.9 (± 17.9)	0.498
BMI, kg/m2	27.1 (± 5.1)	27.4 (± 5)	0.764
Gender, n (%)			
Male	144 (71.6)	13 (39.4)	**0.001**
Female	57 (28.4)	20 (60.6)	
Diabetes mellitus, n (%)			
No	159 (79.1)	28 (84.8)	0.445
Yes	42 (20.9)	5 (15.2)	
ASA score, n (%)			
ASA-1	98 (48.8)	13 (39.4)	**0.002**
ASA-2	97 (48.3)	13 (39.4)	
ASA-3	6 (3)	7 (21.2)	
Surgery side, n (%)			
Right side	110 (54.7)	19 (57.6)	0.760
Left side	91 (45.3)	14 (42.4)	
Pre-op urinary diversion, n (%)			
No	162 (80.6)	24 (72.7)	0.079
Double-J stent	30 (14.9)	4 (12.1)	
Nephrostomy	9 (4.5)	5 (15.2)	
Number of stones	2 (1–3)	3 (1–4)	**0.024**
Multiple stones, n (%)			
No	83 (41.3)	9 (27.3)	0.126
Yes	118 (58.7)	24 (72.7)	
Largest stone size, mm	25.2 (21.5–31.9)	23 (20–30)	0.325

Continuous variables were compared using the independent samples t-test or Mann–Whitney U test based on distribution. Categorical variables were analyzed using the chi-square test or Fisher’s exact test where appropriate. A p value < 0.05 was considered statistically significant

*UTI* urinary tract infection, *BMI* body mass index, *ASA* American Society of Anesthesiologists, *DJ stent* double-J stent, *Fr* French, *Pre-op* preoperative, *Post-op* postoperative

**Fig. 1:**
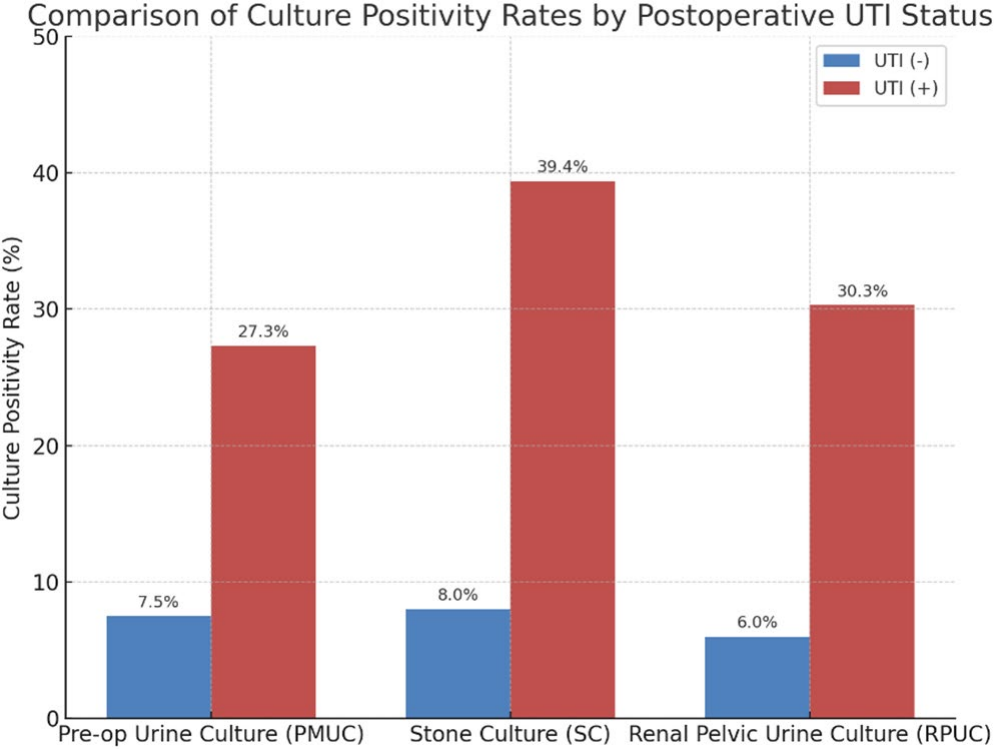
Bar chart illustrating the UTI-positive patients showed significantly higher culture positivity rates than UTI-negative patients in all three methods (PMUC: 27.3% vs. 7.5%, SC: 39.4% vs. 8.0%, RPUC: 30.3% vs. 6.0%; *p* < 0.05). Intraoperative cultures, especially SC, showed higher correlation with postoperative UTI

The groups were comparable with respect to Amplatz sheath size, use of flexible ureteroscopy (ECIRS), urinary drainage method, Foley catheter use, operation time (OT), and stone-free rates (*p* > 0.05). However, the length of hospital stay was significantly longer in the UTI-positive group (8 [7–10] vs. 2 [2–3] days; *p* < 0.001) (Table 2). In the UTI-positive group, the rates of positive PMUC (27.3% vs. 7.5%; *p* = 0.002), SC (39.4% vs. %8; *p* < 0.001), and RPUC (30.3% vs. 6%; *p* < 0.001) were all significantly higher compared to the UTI-negative group (Fig. 1). Notably, in UTI patients receiving antibiotics upon infectious disease consultation, post-operative urine cultures taken before treatment showed growth in only 15.2% of cases.

**Table 2:** Operative and Postoperative Parameters by UTI Status

Variables	UTI (-) *N* = 201 (85.9%)	UTI (+) *N* = 33 (14.1%)	*P* value
Amplatz sheath size, n (%)			
12/16 Fr	47 (23.4)	5 (15.2)	0.661
16/20 Fr	111 (55.2)	21 (63.6)	
20/24 Fr	35 (17.4)	5 (15.2)	
26/30 Fr	8 (4)	2 (6.1)	
Use of flexible ureteroscopy (ECIRS)			
No	156 (77.6)	29 (87.9)	0.179
Yes	45 (22.4)	4 (12.1)	
Post-op urinary drainage, n (%)			
No	32 (15.9)	5 (15.2)	0.832
Double-J stent	127 (63.2)	19 (57.6)	
Nephrostomy	25 (12.4)	6 (18.2)	
Double-J stent + Nephrostomy	17 (8.5)	3 (9.1)	
Post-op foley catheter placement, n (%)			
No	157 (78.1)	22 (66.7)	0.151
Yes	44 (21.9)	11 (33.5)	
Operation time, min	120 (90–150)	130 (102–180)	0.172
Stone-free Status, n (%)			
Stone-free	165 (82.1)	25 (75.8)	0.698
Single residual fragment	21 (10.4)	4 (12.1)	
Multiple residual fragments	15 (7.5)	4 (12.1)	
Fever, n (%)			
No	195 (97)	9 (27.3)	**0.000**
Yes	6 (3)	24 (72.7)	
Pre-op urine culture positivity, n (%)			
No	186 (92.5)	24 (72.7)	**0.002**
Yes	15 (7.5)	9 (27.3)	
Intra-op stone culture positivity, n (%)			
No	185 (92)	20 (60.6)	**0.000**
Yes	16 (8)	13 (39.4)	
Intra-op renal pelvic culture positivity, n(%)			
No	189 (94)	23 (69.7)	**0.000**
Yes	12 (6)	10 (30.3)	
Hospital stay, days	2 (2–3)	8 (7–10)	**0.000**

Continuous variables were compared using the independent samples t-test or Mann–Whitney U test based on distribution. Categorical variables were analyzed using the chi-square test or Fisher’s exact test where appropriate. A p value < 0.05 was considered statistically significant

*UTI* urinary tract infection, *BMI* body mass index, *ASA* American Society of Anesthesiologists, *DJ stent* double-J stent, *Fr* French, *Pre-op* preoperative, *Post-op* postoperative

There was no statistically significant difference in the presence of multiple stones (69% vs. 59.5%; *p* = 0.329), number of stones (2 [1–4] vs. 2 [1–3]; *p* = 0.177) or the size of the largest stone (26 [21.5–31.5] vs. 25 [21-31.8] mm; *p* = 0.949) between patients with and without positive SC results.

Among patients with positive PMUC, the overall rate of preoperative urinary diversion (double-J stent or nephrostomy) was not significantly different compared with those with negative PMUC (29.2% vs. 19.5%, *p* = 0.268). However, when preoperative urinary diversion was analyzed according to diversion type (no diversion, double-J stent, or nephrostomy), the distribution differed between the groups (*p* = 0.004), with a higher proportion of nephrostomy observed in PMUC-positive patients (20.8% vs. 4.3%), whereas double-J stent placement was less frequent (8.3% vs. 15.2%).

Multivariate logistic regression analysis showed that female gender (OR = 3.71, 95% CI: 1.52–9.06, *p* = 0.004), ASA-3 score (vs. ASA-1; OR = 5.13, 95% CI: 1.19–22.18, *p* = 0.029), positive intra-operative SC (OR = 5.83, 95% CI: 1.99–17.03, *p* = 0.001), and RPUC (OR = 3.67, 95% CI: 1.20–11.27, *p* = 0.023) were identified as independent predictors of post-operative UTI (Table 3).

**Table 3:** Independent Predictors of Postoperative UTI

Gender (Female)	OR (Exp(B))	95% CI	*p* value
	3.71	1.52–9.06	0.004
ASA-3 (vs. ASA-1)	5.13	1.19-22-18	**0.029**
Presence of pre-op urinary diversion	0.95	0.339–2.68	0.927
Stone number	1.2	0.94–1.53	0.149
Use of flexible ureteroscopy (ECIRS)	0.53	0.14–1.96	0.338
Operation time	1.002	0.99–1.01	0.548
Post-op foley catheter placement (yes)	2.14	0.79–5.78	0.134
Pre-op urine culture (PMUC) positivity	1.32	0.4–4.41	0.65
Intra-op stone culture (SC) positivity	5.83	1.99–17.03	**0.001**
Intra-op renal pelvic culture (RPUC) positivity	3.67	1.2-11.27	**0.023**

Multivariate logistic regression was performed for variables with *p* < 0.25 in univariate analysis. Statistically significant variables are shown as *p* < 0.05

*UTI* urinary tract infection, *OR* odds ratio, *CI* confidence interval, *Pre-op* preoperative, *Intra-op* intraoperative

## Discussion

PCNL remains the first-line treatment for staghorn and large renal calculi [1]. Despite its minimally invasive nature, it is associated with complications such as bleeding, infection, sepsis, and injury to adjacent organs [11]. Among these, sepsis stands out as a major cause of morbidity and mortality in stone disease [12]. Infectious complications can still occur in patients with sterile PMUC under antibiotic prophylaxis in PCNL [7]. Bacterial colonization within large kidney stones is believed to increase the risk of infection after surgery [7, 13]. Therefore, several studies have investigated whether RPUC and SC are more effective than PMUC in predicting postoperative SIRS or sepsis.

Li et al. reported the incidence of SIRS and urosepsis following PCNL to be approximately 21% and 6%, respectively. They also found SC positivity (21%) to be higher than PMUC (16%) and RPUC (10%) positivity [9]. Mariappan et al. reported SC positivity in 35.2% of cases, RPUC in 20.4%, and PMUC in 11.1% [4]. In our study, the incidence of UTI was 14.1%, and SC showed the highest culture positivity rate among UTI-positive patients (SC: 39.4%, RPUC: 30.3%, PMUC: 27.3%). Notably, SC and RPUC growth was also detected in some patients without postoperative UTI (SC: 8%, RPUC: 6%).

The predictive value of different culture types in postoperative UTI has been previously evaluated. Li et al. found SC positivity to be more strongly associated with SIRS than PMUC or RPUC [9]. Patients with positive SC had a fivefold higher risk of postoperative infection (OR:5.03, *p* = 0.002) [14]. SC and RPUC appear to be more strongly associated with postoperative infectious complications compared to PMUC [7]. Patients with infected SC or RPUC have been found to have a fourfold higher risk of urosepsis (*p* = 0.009) [4]. In a meta-analysis by Zhou et al., positive PMUC (OR = 3.16 95%CI 2.11–4.74), RPUC (OR = 5.81, 95%CI 1.75–19.32), and SC (OR = 5.11, 95%CI: 1.46–17.89) were all identified as predictors of post-PCNL infectious complications [8]. Consistent with these findings, our multivariate analysis showed that intraoperative SC (OR = 5.83, *p* = 0.001) and RPUC (OR = 3.67, *p* = 0.023) as independent predictors of postoperative UTI and demonstrated a higher predictive value for infection complications after PCNL compared to PMUC (OR = 1.32, *p* = 0.65) [4, 7]. The lack of statistical significance for PMUC may be attributed to the administration of appropriate pre-operative antibiotic therapy in patients with positive results.

Interestingly, among patients diagnosed with post-operative UTI following infectious disease consultation, only 15.2% had positive growth in urine cultures obtained before the initiation of antibiotic treatments. The low culture positivity rate may be related to previous empirical/ prophylaxis antibiotic use or low bacterial load at the time of sampling. Given the limited utility of post-operative urine cultures in identifying causative pathogens, intra-operative SC and RPUC cultures may provide additional value in guiding earlier and more targeted antimicrobial treatment.

Early intervention is critical in patients with sepsis to reduce morbidity and mortality [15]. Initiation of appropriate antibiotic is essential in patients with UTI. Given the delay in obtaining culture results, intraoperative SC and RPUC may facilitate early targeted treatment and improve outcomes in patients with sepsis [16]. Therefore, it is recommended to routinely obtain SC and RPUC during PCNL procedures to aid in identifying causative pathogens and guiding antimicrobial management [7].

In our cohort, PMUC positivity alone was not considered an indication of infection, and preoperative nephrostomy or double-J stent placement was primarily performed based on the presence of acute urinary obstruction. This clinical approach may explain the absence of significant differences in overall urinary diversion rates between PMUC-positive and PMUC-negative patients. The literature reports conflicting findings on this issue. Some studies have suggested that preoperative nephrostomy in patients with PMUC may reduce severe infectious complications [17]. Similarly, a retrospective study reported that in high-risk patients, preoperative nephrostomy with RPUC-guided antibiotic therapy combined with intraoperative SC assessment may reduce postoperative UTI rates compared with PMUC-based management alone [18]. In contrast, other studies have shown that preoperatively placed nephrostomy has no significant impact on complication rates or surgical outcomes in PCNL [19]. Moreover, a prospective randomized study comparing percutaneous nephrostomy and double-J stent placement for preoperative drainage in patients with stone-related acute kidney injury found no difference in postoperative infectious complications between the two approaches after stone surgery [20].

In the present study, although PMUC status did not influence the overall need for urinary diversion, nephrostomy was more frequently preferred in PMUC-positive patients. This finding should be interpreted with caution, as it may reflect physician preference in the presence of an infectious clinical profile and may also be influenced by the limited number of patients in the subgroup analysis. Further large-scale, multicenter prospective studies are needed to better clarify the role of preoperative urinary diversion in this setting.

Culture positivity is an independent risk factor for infectious complications after PCNL. Thus, ensuring sterile urine before surgery and administering appropriate antibiotics in cases where sterility cannot be achieved is crucial [1]. However, various patient-related, stone-related or surgical parameters were also evaluated for infective complications after PCNL and conflicting results are observed in the literature.

The role of gender in post-PCNL infections remains controversial. While some studies report no significant association between gender and infection risk [9, 21, 22], Değirmenci et al. observed a higher SIRS rate in male patients [23]. However, female patients are more likely to have positive urine cultures than males (42.9% vs. 21.5%, *p* < 0.01) [24]. Zhu et al. reported a possible association between female sex and increased rates of urosepsis [5]. Similarly, Zhou et al. reported that female gender increased the risk of infectious complications by more than 1.5 times (OR = 1.60, 95% CI 1.23–2.07) [8]. Our study also showed that the proportion of female patients was significantly higher in the postoperative UTI group (60.6% vs. 28.4%, *p* = 0.001), and multivariate analysis confirmed that female gender is an independent predictor of postoperative UTI (OR = 3.71, *p* = 0.004).

Stone burden may be associated with an increased risk of urosepsis. While Rivera et al. found no significant correlation between stone size and postoperative infections, they reported a higher incidence of UTI in patients with multiple stones [21]. In our study, patients in the postoperative UTI group had a higher median stone number (3 [IQR 1–4] vs. 2 [IQR 1–3], *p* = 0.024); however, stone size did not differ significantly between the groups, and multivariate analysis did not identify stone number as an independent risk factor for postoperative UTI.

If correlated with intraoperative SC, stone burden could help predict postoperative urinary tract infections. However, no significant association was observed between stone burden indicators including stone size, number, presence of multiple stones and intraoperative SC positivity.

The association between OT and infection is also debated. Some studies report no significant relationship [9, 21], while others have identified OT as an independent risk factor [5, 8, 25]. In our study, the median OT was slightly longer in the UTI group (130 [IQR 102–180] vs. 120 [IQR 90–150] minutes); however, this difference was not statistically significant (*p* = 0.172), and OT was not identified as an independent predictor in the multivariate analysis. In line with earlier studies, our findings revealed no significant association between age, BMI, or diabetes and the development of postoperative UTI [9, 21, 25].

Increased intrarenal pressure during PCNL may lead to systemic bacterial translocation through tubular, lymphatic, and venous backflow [13]. Smaller tract sizes may be associated with higher intrapelvic pressures and increased risk of postoperative infection [6]. However, we found no significant association between Amplatz sheath size and infectious complications.

A randomized prospective study by Sener et al. found no difference in infection parameters between patients with and without a postoperative foley catheter following ureterorenoscopy [26]. However, to our knowledge, there are no high-level evidence studies evaluating the impact of postoperative foley catheter use on infections after PCNL. In our study, postoperative Foley catheter placement was more common in the group with UTI (33.5% vs. 21.9%), but this difference was not statistically significant (*p* = 0.151) and was not identified as an independent predictor in multivariate analysis.

### Study limitations

This study has several limitations. Its retrospective design may have introduced selection and information bias. Although our sample size is within the acceptable range of similar studies evaluating preoperative and intraoperative cultures in PCNL, it still represents a relatively limited number of cases. The analysis was conducted at a single tertiary referral center, which may limit the generalizability of the findings to other institutions or populations with different microbiological flora or surgical protocols. The lack of standardized assessment of intrarenal pressure and irrigation volume during surgery represents another limitation, as these factors may affect bacterial translocation. Although we used multivariate logistic regression to adjust for confounding factors, unmeasured variables such as stone composition, antibiotic resistance profiles, or intraoperative irrigation pressures may have influenced the outcomes.

## Conclusions

Intraoperative stone and renal pelvic urine culture demonstrated superior predictive value for postoperative urinary tract infection after supine percutaneous nephrolithotomy compared with preoperative midstream urine culture. Female sex and higher ASA scores were identified as independent risk factors for postoperative infection. In UTI-positive patients, pathogen identification was also more limited in postoperative urine cultures obtained before antibiotic therapy, particularly when compared with intraoperative cultures. Routine use of intraoperative cultures may facilitate earlier identification of causative organisms and enable rapid initiation of targeted antibiotic therapy, thus potentially reducing the risk of infectious complications and improving patient outcomes. These findings support routine use of intraoperative cultures in all supine PCNL patients.

## Data Availability

The datasets, materials, and/or code generated and/or analyzed during the current study are not publicly available. However, they may be made available from the corresponding author on reasonable scientific request due to valid academic or ethical considerations.
